# Predicting the Presence of Intra-abdominal Adhesions in Pregnant Women Undergoing a Repeat Cesarean Section by Assessing the Ultrasound Sliding Sign: A Prospective Observational Study

**DOI:** 10.7759/cureus.83645

**Published:** 2025-05-07

**Authors:** Nallaballe Shalini, Shailaja Bidri, Ravi Kumar Yeli, Rajasri G Yaliwal, Aruna Biradar, Preeti S Malapure

**Affiliations:** 1 Department of Obstetrics and Gynaecology, Shri BM Patil Medical College Hospital and Research Centre, Bijapur Lingayat District Educational Association (BLDE) (Deemed to be University), Vijayapura, IND; 2 Department of Radiology, Shri BM Patil Medical College Hospital and Research Centre, Bijapur Lingayat District Educational Association (BLDE) (Deemed to be University), Vijayapura, IND

**Keywords:** cesarean section, intra-abdominal adhesions, maternal outcomes, non-invasive diagnostics, repeat cs, ultrasound sliding sign

## Abstract

Background

Cesarean section (CS) is a common obstetric procedure, with repeat CS increasing the risk of intra-abdominal adhesions, leading to surgical complications. The ultrasound sliding sign has been proposed as a non-invasive tool to predict adhesions, but its diagnostic accuracy remains under evaluation. This study aims to assess the accuracy of the ultrasound sliding sign in predicting intra-abdominal adhesions in repeat CS cases and evaluate its correlation with intraoperative findings.

Methodology

A prospective observational study was conducted at BLDE (D.U.) Shri B.M. Patil Medical College from May 2023 to December 2024, including 200 women undergoing repeat CS. Preoperative ultrasound evaluated the sliding sign, classifying cases as positive (free uterine movement, no adhesions) or negative (restricted movement, adhesions). Intraoperative findings were recorded for correlation. Statistical analysis was performed using SPSS version 26 (IBM Corp., Armonk, NY, USA).

Results

Among the participants, 40% had adhesions. The ultrasound sliding sign demonstrated 86.1% sensitivity, 85.8% specificity, 77.5% positive predictive value, and 91.6% negative predictive value. Adhesions were significantly associated with maternal age (>30 years), parity (≥2), higher body mass index, and multiple previous CSs.

Conclusions

The ultrasound sliding sign is a reliable, non-invasive tool for predicting intra-abdominal adhesions in repeat CS, aiding in surgical preparedness. Given its high diagnostic accuracy, it can enhance clinical decision-making; however, further studies are needed to refine its application.

## Introduction

Cesarean section (CS) is one of the most commonly performed obstetric surgeries worldwide. With the increasing rates of CS, the number of women undergoing repeat CS has also risen significantly [1]. Both immediate and long-term complications are associated with CS, including excessive bleeding, bladder and intestinal injuries, infections, postoperative ileus, and the formation of intra-abdominal adhesions [2]. Adhesions are bands of fibrous tissue that form between organs and surrounding structures, leading to complications such as chronic pelvic pain, infertility, bowel obstruction, and difficulties during subsequent surgeries [3,4]. The severity of adhesions is influenced by factors such as the number of previous CS procedures, the type of surgical technique used, and the nature of postoperative healing [5].

Intra-abdominal adhesions can complicate repeat CS by making surgical access to the uterus more challenging, increasing the risk of unintended injuries to adjacent organs such as the bladder and intestines [5]. Severe adhesions can extend to involve the lower uterine segment, making it difficult for the surgeon to safely incise the uterus and extract the fetus. The process of adhesiolysis, i.e., surgically cutting through adhesions to free tissues, carries the risk of further damage to surrounding organs and excessive bleeding, thereby increasing maternal morbidity [6]. Given these risks, preoperative identification of intra-abdominal adhesions can be of great benefit, allowing surgeons to anticipate complications and adjust their surgical approach accordingly.

Currently, ultrasonography is emerging as a valuable non-invasive diagnostic tool for predicting intra-abdominal adhesions before surgery. One of the most promising sonographic techniques for this purpose is the ultrasound sliding sign, which evaluates the mobility of the uterus relative to the anterior abdominal wall during deep respiration. The sliding sign is observed when the uterus moves freely in relation to the surrounding peritoneal structures. When adhesions are present, this movement is restricted or absent, indicating the presence of fibrous bands anchoring the uterus to adjacent structures [7].

Although the sliding sign has been studied as a predictive tool for intra-abdominal adhesions, its diagnostic accuracy and clinical utility remain areas of ongoing investigation. While some studies suggest a strong correlation between the absence of the sliding sign and the presence of adhesions, others indicate that the sign alone may not be sufficiently reliable as a standalone predictor [8].

Baron et al. demonstrated that the ultrasound sliding sign is a reliable preoperative marker for predicting intra-abdominal adhesions in pregnant women undergoing repeat CSs. Their study found a strong correlation between the absence of the sliding sign and adhesion formation, highlighting its value in surgical preparedness [9]. Similarly, Limperg et al. conducted a systematic review and concluded that the sliding sign has a high negative predictive value (NPV), meaning that, when present, the likelihood of adhesions is low, making it a useful tool for guiding safe surgical entry [10]. However, conflicting evidence exists, as Shu reported that while the sliding sign could predict adhesions in some cases, it failed to detect dense adhesions in nearly half of the patients. This study suggested that although the sliding sign has moderate sensitivity and specificity, additional imaging techniques or clinical risk assessments may be necessary to enhance diagnostic accuracy [11].

These inconsistencies in research findings highlight the need for further studies to validate the clinical applicability of the ultrasound sliding sign in predicting intra-abdominal adhesions before repeat CS. The research gap lies in establishing standardized imaging protocols, improving the accuracy of the sliding sign, and determining how it can be integrated with other diagnostic modalities to enhance surgical preparedness.

This study aims to evaluate the diagnostic accuracy of the ultrasound sliding sign in predicting intra-abdominal adhesions in women undergoing repeat CS. It aims to assess the correlation between preoperative ultrasound findings and intraoperative surgical observations, determining the sensitivity, specificity, positive predictive value (PPV), and NPV of the sliding sign. The study will identify clinical scenarios where this method enhances surgical preparedness and maternal outcomes while contributing to the refinement of ultrasound-based prediction techniques for safer obstetric surgeries.

## Materials and methods

This prospective observational study was conducted in the Department of Obstetrics and Gynecology at BLDE (Deemed to be University), Shri BM Patil Medical College Hospital and Research Centre, Vijayapura. As a tertiary care center, the hospital provided an ideal setting for investigating the correlation between preoperative ultrasound findings and intraoperative surgical assessment of intra-abdominal adhesions in women undergoing repeat lower-segment cesarean section (LSCS). The study was conducted in accordance with the ethical principles of the Declaration of Helsinki, and ethical clearance was obtained from the Institutional Ethics Committee (approval number: BLDE(DU)/IEC/895/2022-23). Written informed consent was obtained from all participants before enrolment.

The study was conducted over 20 months from May 2023 to December 2024. A total of 200 pregnant women who met the inclusion criteria were enrolled during routine antenatal visits or upon admission for elective LSCS. Participants were between 18 and 35 years of age, at least 37 weeks of gestation, and had a history of one or more previous LSCS. Patients undergoing emergency CSs, those with known collagen or tissue disorders, and those with prior abdominal surgeries unrelated to LSCS were excluded from the study to eliminate confounding factors.

The sample size was calculated using the following formula: n = (Z × σ/d)², where Z was 1.96 (Z-score for 95% confidence interval), σ was 2.7 (standard deviation), and d was 0.5 (margin of error). Based on this calculation, a minimum of 200 participants was determined to be statistically sufficient to meet the objectives of the study.

Eligible participants underwent a standardized preoperative ultrasound examination using the sliding sign technique to assess for intra-abdominal adhesions. A 5 MHz abdominal convex probe was used for the evaluation. The probe was placed perpendicular to the previous cesarean scar and moved in multiple directions, namely, vertical, horizontal, and lateral, while the patient was instructed to perform deep breathing maneuvers. This technique facilitated the visualization of the uterine movement relative to the anterior abdominal wall.

The sliding sign was assessed using a standardized technique during transvaginal ultrasonography. It was defined as positive when the anterior rectal wall glided freely against the posterior aspect of the cervix and posterior vaginal wall with gentle pressure applied using the transvaginal probe, indicating the absence of adhesions. A negative sliding sign was recorded when this gliding movement was restricted or absent, suggesting the presence of adhesions. To minimize observer variability, the assessment was performed by experienced sonographers using a uniform protocol. Furthermore, all scans were performed on high-resolution ultrasound machines of similar quality to reduce machine-dependent variability. These preoperative findings were documented and later compared with intraoperative observations made during LSCS to evaluate the diagnostic accuracy of the ultrasound sliding sign technique.

All collected data were entered into Microsoft Excel (Microsoft Corp., Redmond, WA, USA) and subsequently analyzed using SPSS Software version 26 (IBM Corp., Armonk, NY, USA). Descriptive statistics were used to summarize demographic and clinical variables. Inferential statistical analyses were performed using the chi-square test to assess associations between categorical variables. A p-value of less than 0.05 was considered statistically significant.

## Results

**Figure 1:**
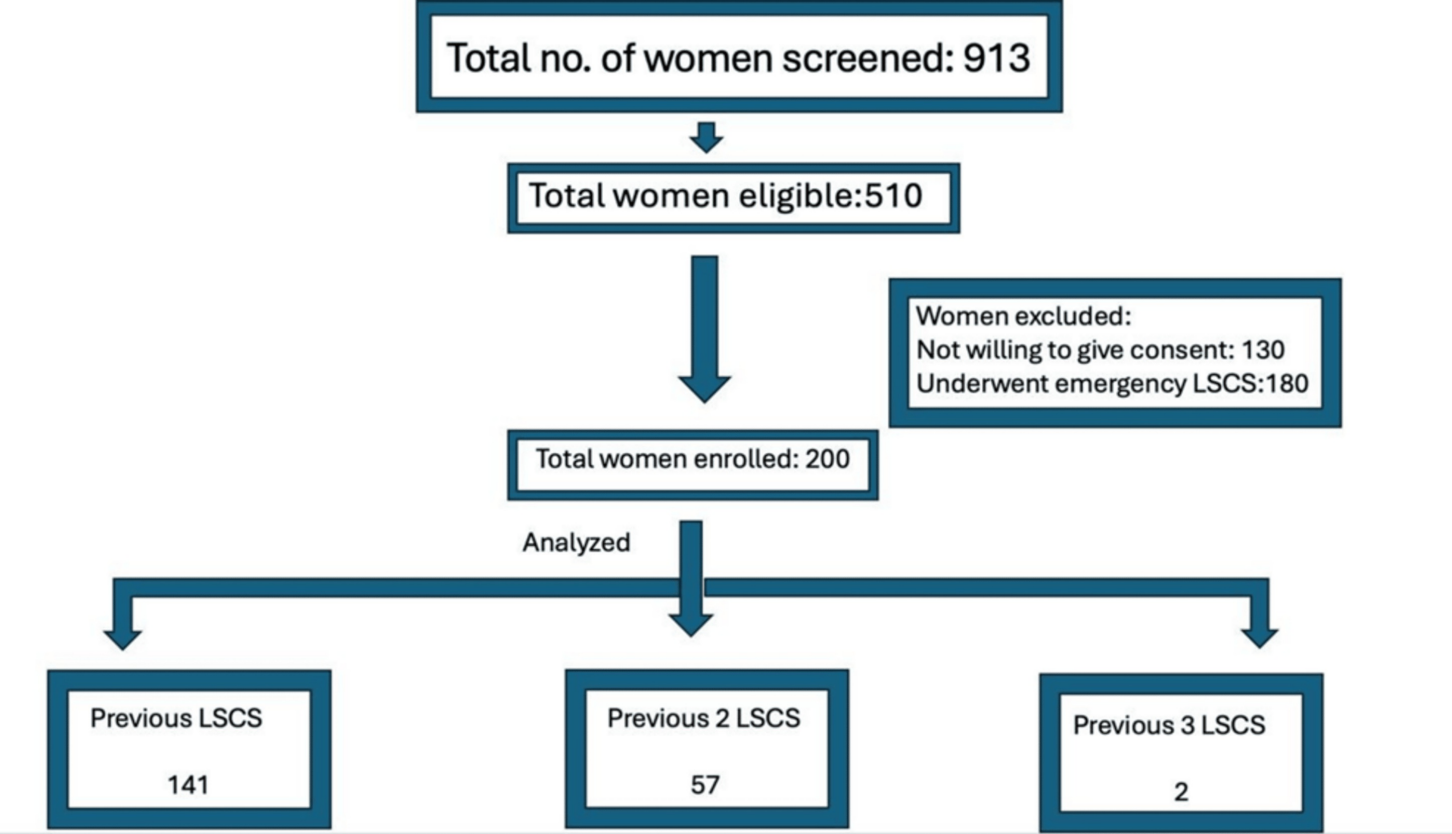
Study flowchart showing patient selection. LSCS: lower-segment cesarean section

A total of 200 pregnant women with a history of previous LSCS were enrolled in the study. Figure 1 presents the study flowchart. The baseline characteristics of the participants are shown in Table 1. The majority, 115 (57.5%), were between 25 and <30 years of age, followed by 57 (28.5%) who were <25 years old, and 28 (14.0%) who were >30 years old. Regarding parity, 125 (62.5%) had a parity of 1, 65 (32.5%) had a parity of 2, 7 (3.5%) had a parity of 3, and 3 (1.5%) had a parity >4. A total of 141 (70.5%) had one previous LSCS, 57 (28.5%) had two, and 2 (1.0%) had three. Regarding body mass index (BMI), 137 (68.5%) had normal BMI, 55 (27.5%) were overweight, and 8 (4.0%) were obese (Table 1).

**Table 1:** Baseline characteristics of the study population (N = 200). Frequency and percentages were calculated for all variables

Variable	Category	Frequency (n)	Percentage (%)
Maternal age	<25 years	57	28.5
	25 to <30 years	115	57.5
	>30 years	28	14.0
Parity	1	125	62.5
	2	65	32.5
	3	7	3.5
	>4	3	1.5
Previous cesarean section	1	141	70.5
	2	57	28.5
	3	2	1.0
Body mass index	Normal	137	68.5
	Overweight	55	27.5
	Obese	8	4.0

Intra-abdominal adhesions were present in 80 (40.0%) participants, while 120 (60.0%) had no adhesions based on intraoperative assessment (Table 2).

**Table 2:** Distribution of intra-abdominal adhesions. Frequency and percentages were calculated for all variables.

Intra-abdominal adhesion	Frequency	Percent
No adhesions	120	60.0
Adhesions	80	40.0
Total	200	100.0

**Figure 2:**
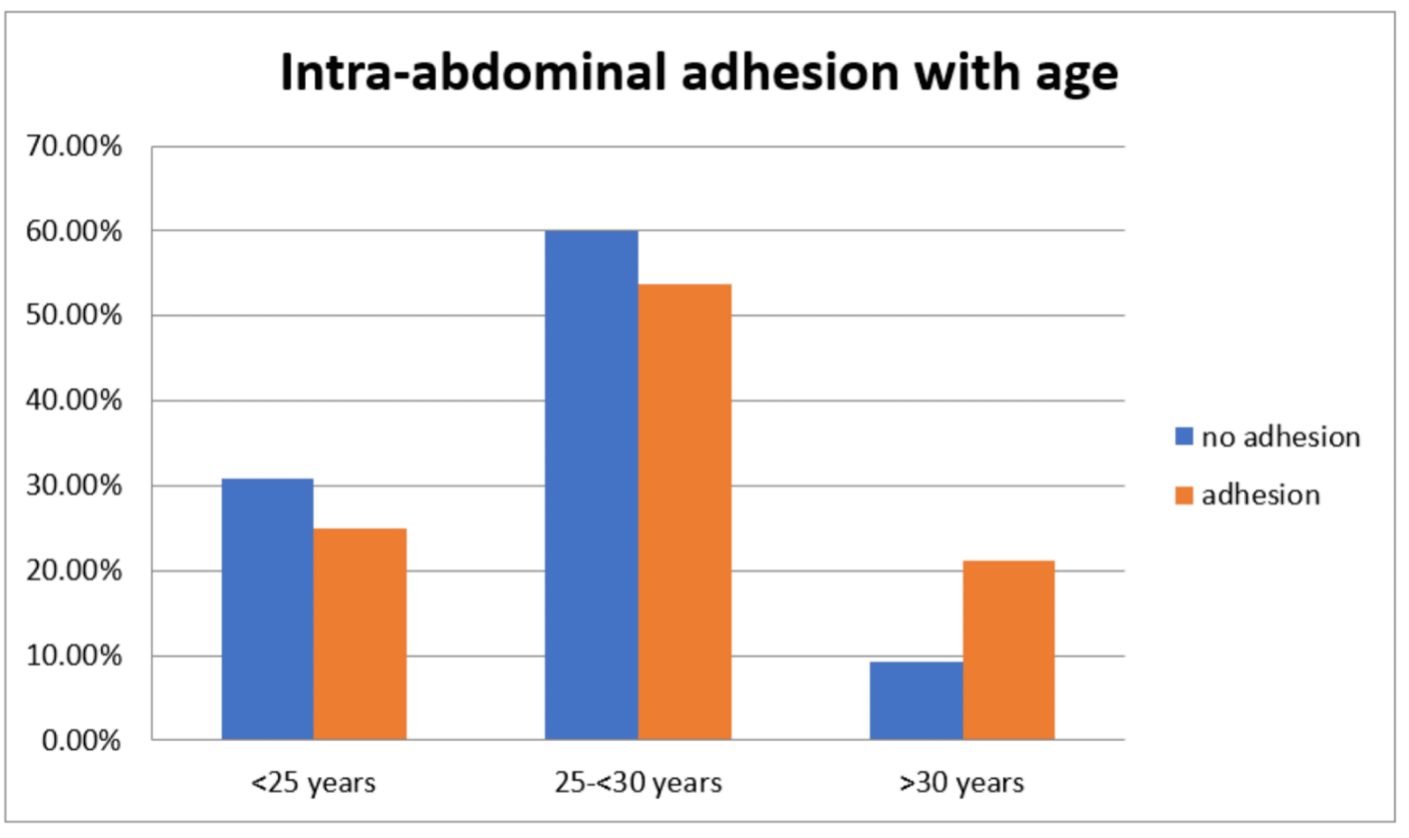
Distribution of maternal age with intra-abdominal adhesions.

The distribution of maternal age in relation to intra-abdominal adhesions revealed that, among 120 (100%) participants without adhesions, 72 (60.0%) were between 25 and <30 years old, 37 (30.8%) were <25 years old, and 11 (9.2%) were >30 years old. In contrast, among 80 (100%) participants with adhesions, 43 (53.8%) were between 25 and <30 years old, 20 (25.0%) were <25 years old, and 17 (21.2%) were >30 years old. The association between maternal age and adhesion formation was statistically significant (Figure 2).

A strong correlation was also observed between parity and intra-abdominal adhesion. Among the 120 (100%) women without adhesions, 90 (75.0%) had a parity of 1, 28 (23.3%) had a parity of 2, and 2 (1.7%) had a parity of 3. None had parity >4. Among 80 (100%) participants with adhesions, 35 (43.8%) had a parity of 1, 37 (46.2%) had a parity of 2, 5 (6.2%) had a parity of 3, and 3 (3.8%) had a parity >4. The difference was statistically significant (p < 0.001) (Table 3).

**Table 3:** Distribution of parity with intra-abdominal adhesions. Frequency and percentages were calculated for all variables. The chi-square test was applied, and a p-value less than 0.05 was considered significant.

Parity	Intra-abdominal adhesions		Total	Chi-square value	P-value
	No adhesions	Adhesions			
1	90	35	125	22.637	<0.001
	75.0%	43.8%	62.5%		
2	28	37	65		
	23.3%	46.2%	32.5%		
3	2	5	7		
	1.7%	6.2%	3.5%		
>4	0	3	3		
	0.0%	3.8%	1.5%		
Total	120	80	200		
	100.0%	100.0%	100.0%		

**Figure 3:**
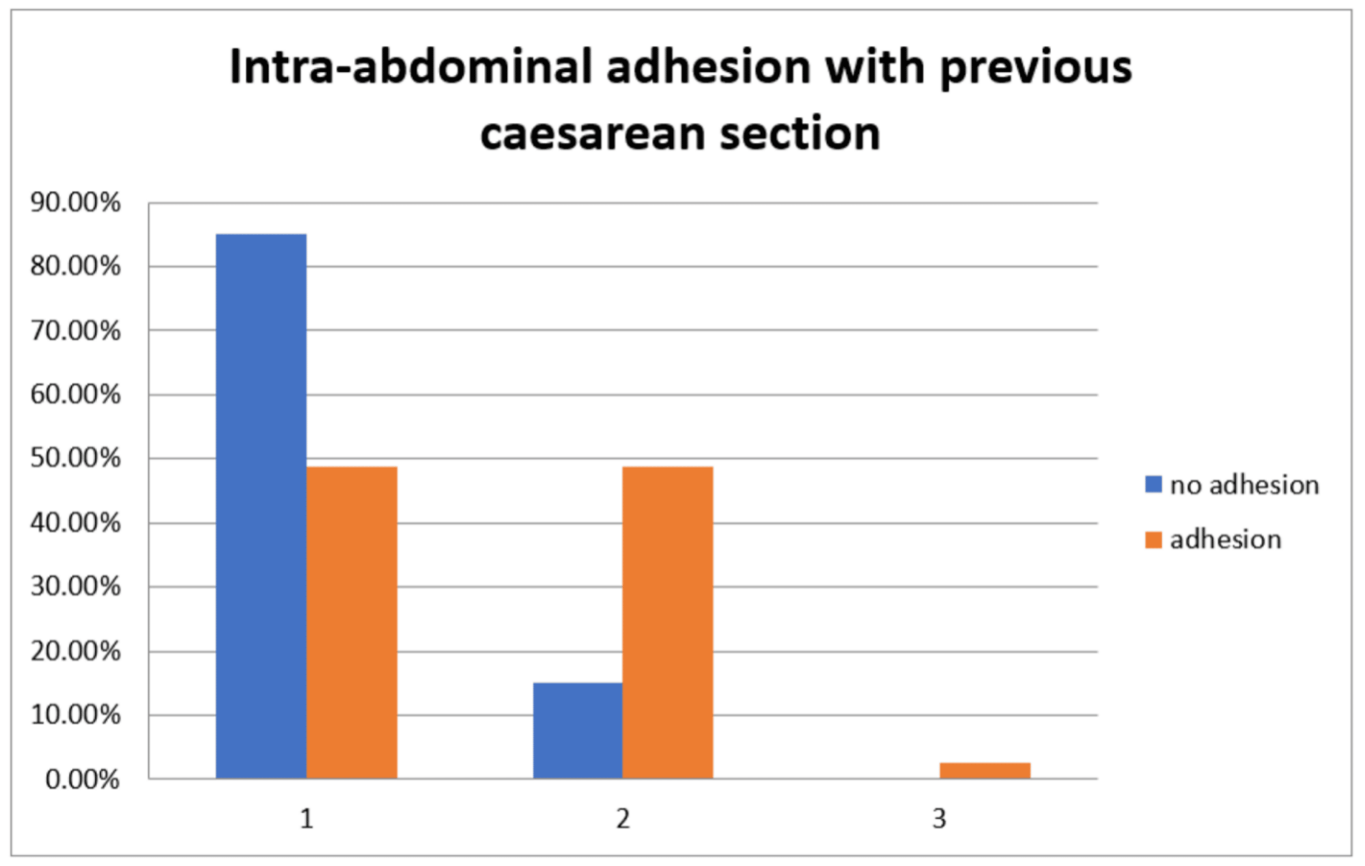
Distribution of previous cesarean sections with intra-abdominal adhesions.

Regarding the number of previous CSs, among the 120 (100%) participants without adhesions, 102 (85.0%) had one LSCS, 18 (15.0%) had two LSCSs, and none had three. Among 80 (100%) participants with adhesions, 39 (48.8%) had one LSCS, 39 (48.8%) had two LSCSs, and 2 (2.5%) had three LSCSs. Although the number of women with three previous LSCSs was small, a striking 5 out of 7 (71.4%) in this subgroup had adhesions, highlighting a strong trend. The association was statistically significant (Figure 3).

**Figure 4:**
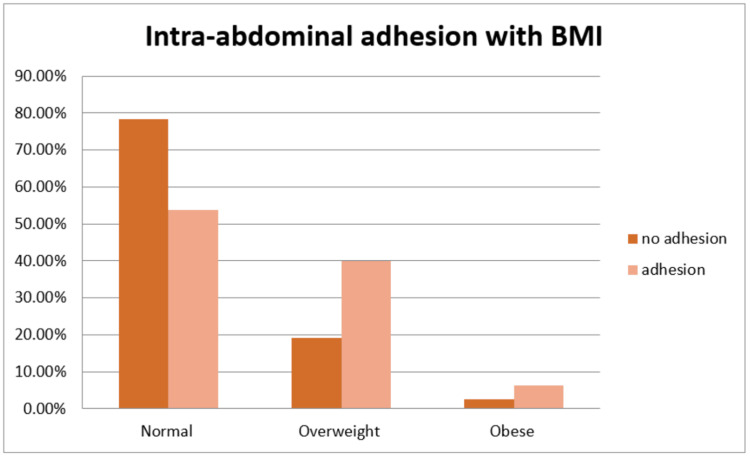
Distribution of body mass index (BMI) with intra-abdominal adhesions.

The association between BMI and intra-abdominal adhesions was also notable. Of the 120 (100%) participants without adhesions, 94 (78.3%) had a normal BMI, 23 (19.2%) were overweight, and 3 (2.5%) were obese. Among the 80 (100%) participants with adhesions, 43 (53.8%) had a normal BMI, 32 (40.0%) were overweight, and 5 (6.2%) were obese. This association was statistically significant (Figure 4).

The ultrasound sliding sign was positive in 128 (64.0%) participants and negative in 72 (36.0%) participants (Table 4).

**Table 4:** Distribution of the sliding sign. Frequency and percentages were calculated for all variables.

Sliding sign	Frequency	Percent
Positive	128	64.0
Negative	72	36.0
Total	200	100.0

Among the 128 (100%) participants with a positive sliding sign, 110 (85.9%) had no adhesions, and 18 (14.1%) had adhesions. Conversely, among the 72 (100%) participants with a negative sliding sign, 10 (13.9%) had no adhesions, while 62 (86.1%) had adhesions. This association was highly significant (p < 0.001). The diagnostic performance of the sliding sign was as follows: sensitivity, 86.0%; specificity, 86.1%; PPV, 91.7%; and NPV, 77.6% (Table 5).

**Table 5:** Distribution of intra-abdominal adhesions with the sliding sign. Frequency and percentages were calculated for all variables. The chi-square test was applied, and a p-value less than 0.05 was considered significant.

Intra-abdominal adhesions	Sliding sign		Total	Chi-square value	P-value
	Positive	negative			
No adhesions	110	10	120	99.667	<0.001
	85.9%	13.9%	60.0%		
Adhesions	18	62	80		
	14.1%	86.1%	40.0%		
Total	128	72	200		
	100.0%	100.0%	100.0%		

**Figure 5:**
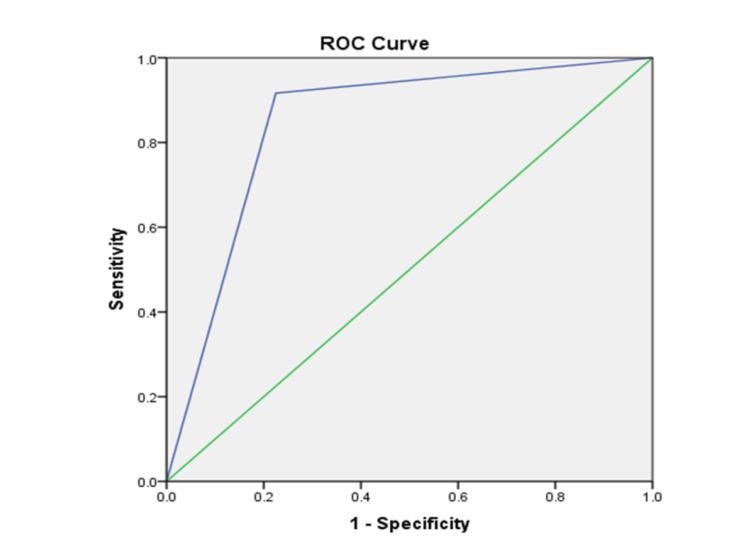
Receiver operating characteristic (ROC) curve analysis for the sliding sign.

The receiver operating characteristic curve for the sliding sign demonstrated an area under the curve of 0.846, indicating good diagnostic accuracy. The upward deflection of the curve toward the upper-left corner in Figure 5 reflects the test’s strong ability to distinguish between the presence and absence of adhesions.

## Discussion

This study assessed the efficacy of the ultrasound sliding sign in predicting intra-abdominal adhesions among women undergoing repeat CSs. Our findings indicated that 40% of the participants had intra-abdominal adhesions, with a significant association between a negative sliding sign and the presence of adhesions. Specifically, 86.1% of women with a negative sliding sign had adhesions, whereas only 14.1% with a positive sliding sign had adhesions. This underscores the potential of the sliding sign as a predictive tool for intra-abdominal adhesions.

Our study revealed a significant correlation between maternal age and the presence of intra-abdominal adhesions. Among participants without adhesions, 30.8% were under 25 years old, 60% were between 25 and 30 years old, and 9.2% were over 30 years old. Conversely, among those with adhesions, 25% were under 25 years old, 53.8% were between 25 and 30 years old, and 21.2% were over 30 years old. This suggests that older maternal age is associated with a higher likelihood of adhesion formation. These findings align with previous research by Alpay et al., indicating that advanced maternal age may contribute to increased adhesion formation due to factors such as reduced tissue elasticity and altered healing processes [12].

Parity was found to be significantly associated with the presence of adhesions. Among women without adhesions, 75% had a parity of 1, 23.3% had a parity of 2, and 1.7% had a parity of 3. In contrast, among those with adhesions, 43.8% had a parity of 1, 46.2% had a parity of 2, and 6.2% had a parity of 3. This indicates that higher parity is associated with an increased risk of adhesion formation. Similar observations were reported by Gurol-Urganci et al., suggesting that repeated pregnancies and deliveries may contribute to the development of adhesions due to cumulative tissue trauma and healing responses [13].

The number of previous CSs was also significantly associated with adhesion formation. Among participants without adhesions, 85% had one previous CS, and 15% had two previous CSs. Among those with adhesions, 48.8% had one previous CS, 48.8% had two previous CSs, and 2.5% had three previous CSs. This suggests that an increasing number of CS procedures correlates with a higher risk of adhesion development. This finding is consistent with Tulandi et al., indicating that each subsequent CS increases the likelihood of adhesion formation, which can complicate future abdominal surgeries [14].

BMI was found to have a significant association with adhesion presence. Among women without adhesions, 78.3% had a normal BMI, 19.2% were overweight, and 2.5% were obese. In contrast, among those with adhesions, 53.8% had a normal BMI, 40% were overweight, and 6.2% were obese. This indicates that higher BMI is associated with an increased risk of adhesion formation. Obesity has been linked to altered wound healing and increased inflammatory responses, which may contribute to adhesion development [15].

The ultrasound sliding sign demonstrated high diagnostic accuracy in predicting intra-abdominal adhesions. Among participants with a positive sliding sign, 85.8% had no adhesions, while 14.1% had adhesions. Conversely, among those with a negative sliding sign, 13.9% had no adhesions, and 86.1% had adhesions. This high sensitivity and specificity highlight the utility of the sliding sign as a non-invasive preoperative assessment tool. Similar findings have been reported by Charernjiratragul et al., supporting the use of the sliding sign in predicting adhesions and aiding in surgical planning to minimize complications [16].

This prospective observational study is strengthened by its standardized methodology, adequate sample size, and direct correlation of preoperative ultrasound findings with intraoperative observations, enhancing the reliability of results. The high sensitivity and specificity of the ultrasound sliding sign support its utility as a non-invasive predictive tool for intra-abdominal adhesions in repeat CSs. However, limitations include its single-center design, which may affect generalizability. Further multicentric studies with broader population diversity are recommended to validate and refine its clinical applicability.

## Conclusions

Our study reinforces the utility of the ultrasound sliding sign as a valuable preoperative tool for predicting intra-abdominal adhesions in women undergoing repeat CSs. The significant correlation between a negative sliding sign and the presence of adhesions highlights its potential to enhance surgical preparedness and improve maternal outcomes. Integrating the assessment of the sliding sign into routine preoperative evaluations could lead to more informed surgical strategies and reduced intraoperative risks. Future research should focus on standardizing the sliding sign technique and exploring its integration with other diagnostic modalities to further improve the prediction and management of intra-abdominal adhesions.
